# Notes and News

**Published:** 1890-02-15

**Authors:** 


### Notes and News.

Collected and Communicated.

The last annual report of the Princess Alice Hospital says words of praise of the new building, and of the successful manner in which the work was carried out. The public have come forward nobly with funds, and it is shortly hoped to pay off the remaining debt of £500.

A course of four lectures to ladies on Domestic Hygiene will be given at the Sanitary Institute, 74a, Margaret Street, W., commencing on February 24th. The Duchess of Albany has expressed her intention of being present, and of distributing the certificates won at the examination at the close of the course. Tickets, 10s., will admit to all four lectures, or payment can be made at the doors at the rate of 2s. 6d. each admission.

The fifth general meeting of the Hospitals Association will be held in the Board-room of Charing Cross Hospital on February 20th. The chair will be taken by Mr. E. Murray Ind, chairman of the London Hospital, at half-past eight p.m. A paper will then be read by Mr. Henry C. Burdett, on "The Financial Aspects of the Hospital Problem, and How to Remedy some Existing Defects."

At an inquest held at Portsmouth last week it was stated that Daniel Ferbridge, a baker's carter, was seen at the corner of Melbourne Street with the reins in his hand, and as the horse and cart remained in the same position for an hour, the man was examined, and found to be dead. The evidence of his father was that he was suffering from influenza, but could not afford to stay at home. A verdict of death from syncope caused by influenza was returned.

Sheffield was unfortunate in having a very wet day for Hospital Sunday, and thus the collections showed a slight falling-off in some of the churches. The total was £2,050, against £2,174 subscribed the year before. The Church of England gives £1,062, the Roman Catholics £35, the Wesleyans £217, and the Jews £5. At Rotherham, which held Hospital Sunday the same day, £150 was collected, against £245 for the preceding year, but all the returns were not then to hand.

An entertainment was given to the present and several of the past patients and nurses at the Chelmsford Infirmary on January 18th, 1890. A very interesting programme was successfully carried out. The lighting of the several scenes was well arranged, and some capital music was provided by the Misses Copland, Coleman, Lewis, and G. Veley, and by the Rev. G. V. Collier and Mr. Sargent. The whole entertainment was under the management and superintendence of Miss New, the matron, and Miss G. Veley, daughter of the honorary secretary.

The Royal College of Physicians has an excellent system of fire-extinguishing apparatus connected to the constant-pressure water-mains in Pall Mall, enabling several jets to be thrown into any part of the building in case of an outbreak of fire, or indeed to attack any fire occurring in their neighbours' premises. The porters of the college are periodically drilled by one of Messrs. Merryweather and Sons' fire-inspectors, and after each drill the secretary receives a printed report as to the condition of the fire-appliances and the efficiency, or otherwise, of those taking part in the drill.

On January 30th Mrs. James Montague formally opened the new Mexborough Montague Cottage Hospital, in the presence of a large concourse of people. Previous to the ceremony there was a gathering in the new Primitive Methodist Schoolroom, where the chair was occupied by Mr. H. Liversedge, chairman of the Working Men's Committee, which has been mainly instrumental, along with Dr. Sykes, in bringing about the establishment of the institution. Mrs. Montague was presented with a silver key in memory of the ceremony. In the evening a concert was given. The hospital has been opened under good auspices, and is likely to prove successful.

An excellent account of the history and present state of the Norfolk Hospital lately appeared in a local paper. The hospital commenced in 1772 with accommodation for three patients; it now treats 5,000. The hospital has eight wards, four in each pavilion on ground and upper floors, but only six are occupied—three by males, two by females, and one by children. The empty wards are those on the ground floor of the pavilion nearest to the city, above which the female wards are situated. If occupied, they would accommodate at least 45 beds, but the hospital is too poor to bear the expense. We hope the article will so far interest the public in the hospital that they will no longer bear the disgrace of these closed wards.

A curious tale of a high temperature comes from one of our London hospitals. The old wardrobe-keeper of Drury Lane years ago had cancer, and went into hospital for an operation. The operation was performed, and all went well till the following Sunday, when suddenly the old lady's temperature went up, she became restless, and feverish symptoms set in. There seemed no reason for this change for the worse; the wound looked well, and the puzzled surgeon determined to discover the cause of the high temperature at all costs. He entered into conversation with his patient, and soon the hidden evil was brought to light. For many years the old lady had had her *Referee* every Sunday morning regularly, till on this occasion the hospital porter had told her Sunday papers were not allowed. This prohibition had caused the patient much annoyance, and roused the feverish symptoms observed. The house-surgeon promptly went out and bought a *Referee*, and read the old lady the dramatic gossip for which she pined. The temperature went down, the patient became calm, and ultimately made a good recovery. But she had been quite prepared to die of vexation if deprived of her *Referee*.

The following vacancies are announced this week, the amount of the salary in each case being given after the name of the institution :

**Resident Medical Officers.**

North Staffordshire Infirmary. House Physician—£100, board, etc.
Nottingham General Hospital. Medical Assistant—Board, etc.
Newcastle City Hospital. Medical Assistant—£50, board, etc.

**Non-Resident Medical Officers.**

Swindon Union District Medical Officer—£70, Board, etc.

The Hospital for Women, Soho Square. Clinical Assistants.

An able-bodied pauper is an anomaly; he ought not to exist. He does exist, however, but we are glad to say he is becoming less frequent. In 1863, when the number was the highest, there were 9·1 adult able-bodied paupers in every 1,000 of the population, while in 1889 the number of paupers of this class had fallen to 3·0 per 1,000. The number of adult paupers classified as able-bodied, who were in receipt of relief on July 1st, 1889, was smaller than the number relieved on the same day in any of the other years except 1876, notwithstanding the increase in the population. How it would have gladdened the heart of Carlyle to hear that the poor law had fewer able-bodied men shut up within its grasp, and unable or unwilling to work out their own salvation.

The Press has lately been occupying itself greatly with the health of the Navy, of which statistics have just been published. The returns for the total force are the most satisfactory that have been shown since they were first published in their present form in 1856. The total force in the service afloat in 1888 was 50,060 officers and men, and the total number of cases of disease and injury entered on the sick list was 49,439, in the ratio of 987·41 per 1,000, being a decrease compared with the preceding year of 31·4 per 1,000, and a decrease of 126·43 per 1,000 when compared with the average ratios for the last ten years. During the year the lowest sick rate was on the south-east coast of America station, and the highest on the East Indian station. Compared with 1887 there was an increase in the death rate on the south-east coast of America and the East Indies stations, and a reduction on all the other stations.

The Bradford Joint Hospital Fund during the past year (1889) has decidedly been successful, seeing that the amount received has increased something like £1,000 over 1888, and £750 over 1887, the actual figures being—1889, £5,245 13s.; 1888, £4,282 3s. 11d.; 1887, £4,496 11s. 10d. This increase has resulted chiefly, it would appear, through the increased contributions of working people whose £2,319 practically means four-fifths of the total subscriptions received. We may mention, in order to show how actively the employé's committee have worked, that four years ago the amount received from this source was only £800. Fêtes got up in aid of the fund have been a considerable source of revenue, no less than £1,450 being obtained from one, the Peel Park Galas (against £1,050 in the previous year), while the Football Charity cup produced £197, the total amount received from the latter source since the presentation of the cup, five years ago, being no less than £1,084 18s. 3d. Strange to say, however, the income from collections in places of worship have been decreasing during the last few years; but now, however, hopes are entertained that a better revenue will be obtainable for this, as there is a slight increase on last year, the amounts being: 1889, £775 10s. 9d.; 1887-8, £731 17s. 5d. The money received has been disbursed in the following manner: £3,971 has been given to the Infirmary; £641 to the Eye and Ear Hospital; £409 to the Children's Hospital; and, after paying these and defraying expenses, a balance of £1 5s. 8d. left at the bank. Bradford, truly, is to be congratulated on this highly-successful issue of their efforts on behalf of their sick brethren.

The annual meeting of the Leeds Workpeople's Hospital Fund has been held at the Infirmary, Alderman Spark, president, occupying the chair. There were also present: Mr. R. Benson Jowitt, treasurer of the fund; Mr. C. H. Wilson, one of the honorary secretaries; and a large number of members of the committee. Mr. Wilson read the annual report, which expressed gratification at the magnificent success that had been scored during 1889, £5,170 3s. 9d. having been subscribed. The sum of £4,938 3s. 5d. had been divided as follows: Infirmary, 82 per cent., £4,409 6s.; dispensary, 10 per cent., £493 16s. 3d.; Hospital for Women and Children, 8 per cent., £395 1s. 2d. These amounts showed respective increases of £695 8s. 4d., £84 16s., and £67 16s. 9d. The expenses had amounted to £232 0s. 4d., made up as follows: Stationery, printing, &c., £51 16s. 11d.; advertising subscriptions, £147 4s. 10d.; meeting rooms, £7 16s.; wood boxes, £5 6s. 3d.; and postages, £19 16s. 4d. This was £3 19s. less than the expenses of 1888. The workpeople of Leeds deserve the highest commendation.

On January 26th passed peacefully away James E. Adams, aged only 45 years. At the London and Moorfields Hospitals, where he worked from student to senior surgeon, he was beloved by pupils, nurses, and patients, and truly esteemed by his fellow-workers. An honourable, upright Englishman, who truly did his duty to all classes of patients without one sordid thought, devoted to his profession, he who had been the means of saving the sight of hundreds, was some four years ago struck down by blindness. How nobly and patiently he bore this affliction those who knew him in the little country village to which he retired can tell. His nearest ones never heard him murmur, and he was so fearful of giving trouble to those around him that instead of being waited upon he waited on others, and taught himself the thousand and one little things which one only thinks a person blind from his birth can do. He was always ready with the best advice and work for all patients who came to him of whatever class. Patiently he suffered, not only his blindness, but the knowledge of what might at any time follow, knowledge which cannot be kept from a medical man. He has now passed away to the land "where the eyes of the blind shall see," and we print this short record as a word of encouragement to those who are similarly afflicted.

The Ladies' Charity School for Training Young Girls as Domestic Servants, though nearly two hundred years old, was able to introduce a novel idea for an entertainment on the occasion of the prize giving. Colonel Price opened the proceedings with a short speech, in the course of which he said the institution was in great need of funds and new friends to keep up the efficiency, and that the committee proposed to try and raise money after Easter by holding a bazaar. He then thanked Lady Clerke for her kindness in coming to give the children prizes, which ceremony then followed. After songs, a lecture, and tea, the girls and their visitors found that the committee had been able to realize for them that often spoken of but seldom seen, "The good ship come home at last," laden with treasures for everyone present. There it was in full sail, a three-masted vessel of six feet long, on an emerald sea, gaily dressed with flags, and Britannia presiding o'er the waves, surrounded by three noble tars. After singing "Rule Britannia" with a flourish of Union Jacks at the end of each verse, the not least enjoyable part of the performance for the children began, *i.e.*, the unlading of the cargo, when it was found that the same kind friend of former years had remembered the girls of the Ladies' Charity School, and the committee had remembered the little visitors, so that there was something for all taken out of the hold of the ship.

